# Supplementary material for: Uncovering Novel Extracellular Matrix Transcriptome Alterations in Lesions of Multiple Sclerosis
Source: Int J Mol Sci. 2024 Jan 19;25(2):1240. doi: 10.3390/ijms25021240 (PMC10816920; doi:10.3390/ijms25021240)
Supplement: Supplementary file 1 [file ijms-25-01240-s001.zip › ijms-2791481-supplementary.pdf]

Supplemental/Extra Figures

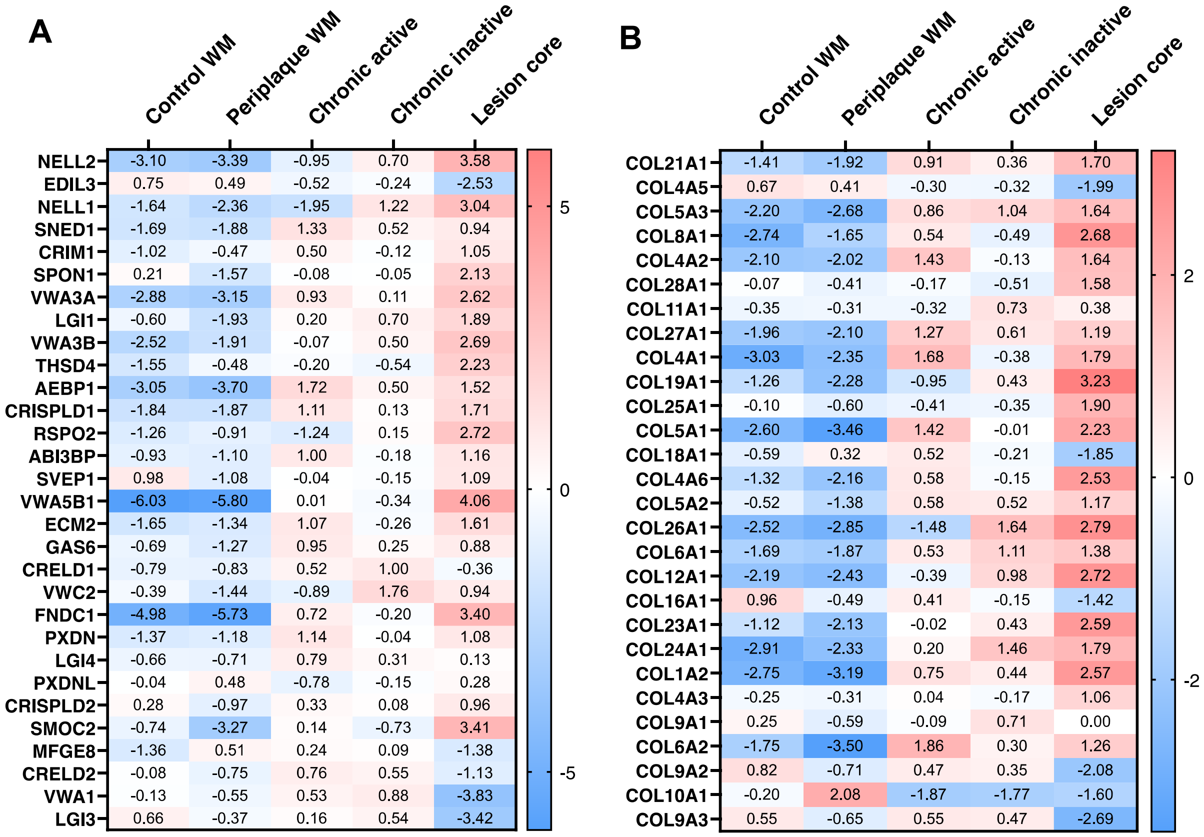

Suppl Figure S1. Greatest differentially expressed proposed glycoproteins and collagens. **(A)** Greatest differentially expressed list of minor glycoproteins. (**B),**Full list of greatest differentially expressed collagen genes. Listed genes are in descending order of greatest median expression in lesion core (top=greatest), have adjusted *p*-value<0.05 log2 fold change, and median-normalized expression > 0.05 in at least one condition.

Supplementary Tables

Supplementary Table S1. Major glycoproteins

Gene names	Protein names and synonyms
**Major known glycoproteins**	
Efemp1,2	Fibulins 3,4/Epidermal growth factor-containing fibulin-like extracellular matrix protein 1,2
Eln	Elastin
Emid1,2	EMI domain containing 1,2
Emilin1-3	Elastin microfibril interfacer 1-3
Fbn1,2	Fibrillin
Fbln1,2,5,7	Fibulin 1,2,5,7
Fn1	Fibronectin 1
Fras1	Fraser extracellular matrix complex subunit/Fraser syndrome homolog 1
Gldn	Gliomedin
Hmcn1,2	Hemicentin 1,2
Ibsp	Integrin binding sialoprotein, BSP
Matn1-4	Matrilin 1-4
Mfap1a,1b, 2-5	Microfibrillar-associated proteins 1A,1B, 2-5
Mmrn1,2	Multimerin 1,2
Npnt	Nephronectin
Papln	Papilin, proteoglycan-like sulfated glycoprotein
Postn	Periostin, osteoblast specific factor
Sparc	Secreted acidic cysteine rich glycoprotein
Sparcl1	Secreted acidic cysteine rich glycoprotein-like 1, hevin
Spp1	Secreted phosphoprotein 1, osteopontin
Srpx	Sushi-repeat-containing protein
Tnc, n, r, xa, xb	Tenascins c, n, r, xa, xb
Thbs1-4	Thrombospondins 1-4
TSP5/Comp	Trhombospondin 5/Cartilage oligomeric matrix protein
**Basement membrane components**	
Colq	Collagen-like tail subunit of asymmetric acetylcholinesterase
Lama1-5	Laminin alpha subunits
Lamb1-4	Laminin beta subunits
Lamc1-3	Laminin gamma subunits
Nid1,2	Nidogens 1,2

**CNS-enriched glycoproteins**	
Agrn	Agrin
Coch	Cochlin
Ntn1-4	Netrins
Ntn1	Netrins G1, G2
Reln	Reelin
Slit1-3	Slit homologs 1-3
Sspo	SCO-spondin
Tecta,b	Tectorins alpha and beta
**Vascular ECM proteins**	
Fga, b, g	Fibrinogen alpha, beta, gamma chains
Vtn	Vitronectin
Vwf	von Willebrand factor
**CCN family proteins**	
CCN1/Cyr 61	Cysteine rich protein 61, CCN1
CCN2/Ctgf	Connective tissue growth factor
CCN3/Nov	Nephroblastoma overexpressed gene
CCN4-6/Wisp1-3	WNT1 inducible signaling pathway proteins
**Growth-factor binding proteins**	
Bmper	BMP-binding endothelial regulator
Igfals	Insulin-like growth factor-binding protein, acid labile subunit
Igfbp1-7	Insulin-like growth factor-binding proteins 1-7
Igfbpl1	Insulin-like growth factor-binding protein-like 1
Kcp	Kielin/chordin-like protein
Ltbp1-4	Latent transforming growth factor beta-binding protein
**ECM of bones, cartilage, and teeth**	
Ambn	Ameloblastin
Amelx	Amelogenin X chromosome
Bglap2	Bone gamma-carboxyglutamate protein 2

Bglap-rs1	Bone gamma-carboxyglutamate protein-related sequence 1
Cilp	Cartilage intermediate-layer protein, nucleotide pyrophosphohydrolase

Cilp2	Cartilage intermediate layer protein 2
Dmp1	Dentin matrix protein 1
Dpt	Dermatopontin
Dspp	Dentin sialophosphoprotein
Mgp	Matrix Gla protein

Supplementary Table S2. Other glycoproteins

**Abi3bp**	ABI gene family, member 3 (NESH)-binding protein
**Adipoq**	Adiponectin, C1Q, and collagen domain-containing protein
**Aebp1**	AE-binding protein 1
**Bsph1**	Binder of sperm protein homolog 1
**Cdcp2**	CUB domain-containing protein 2
**Creld1/2**	Cysteine-rich with EGF-like domains 1 and 2
**Crim1**	Cysteine-rich transmembrane BMP regulator 1 (chordin like)
**Crispld1/2**	Cysteine-rich secretory protein LCCL domain-containing 1 and 2
**Cthrc1**	Collagen triple helix repeat containing 1
**Ddx26b**	DEAD/H (Asp-Glu-Ala-Asp/His) box polypeptide 26B
**Dmbt1**	Deleted in malignant brain tumors 1
**Ecm1,2**	Extracellular matrix protein 1, 2/female organ, and adipocyte specific
**Edil3**	EGF-like repeats and discoidin I-like domains 3
**Egflam**	EGF-like, fibronectin type III, and laminin G domains
**Fgl1/2**	Fibrinogen-like proteins 1 and 2
**Fndc1/7/8**	3 Fibronectin type III domain-containing proteins
**Gas6**	Growth arrest specific 6
**Igsf10**	Immunoglobulin superfamily, member 10
**Lgi1-4**	4 Leucine-rich repeat LGI family proteins
**Lrg1**	Leucine-rich α-2-glycoprotein 1
**Mepe**	Matrix extracellular phosphoglycoprotein with ASARM motif
**Mfge8**	Milk fat globule-EGF factor 8 protein
**Nell1/2**	NEL-like 1 and 2
**Oit3**	Oncoprotein-induced transcript 3
**Otog**	Otogelin
**Pcolce/Pcolce2**	2 Procollagen C-endopeptidase enhancer proteins
**Pxdn**	Peroxidasin homolog
**Rspo1-4**	4 R-spondin homologs
**Slamf6**	SLAM family member 6
**Smoc1,2**	SPARC-related modular calcium-binding proteins 1 and 2
**Sned1**	Sushi, nidogen, and EGF-like domains 1
**Spon1,2**	Spondin 1, (f-spondin) extracellular matrix protein, 2/extracellular matrix protein
**Srpx2**	Sushi-repeat-containing protein, X-linked 2
**Svep1**	Sushi, von Willebrand factor type A, EGF, and pentraxin domain-containing 1
**Tgfbi**	Transforming growth factor β-induced
**Thsd4**	Thrombospondin type I domain-containing 4
**Tinag/Tinagl1**	Tubulointerstitial nephritis antigen/tubulointerstitial nephritis antigen-like 1
**Tnfaip6**	Tumor necrosis factor α-induced protein 6
**Tsku**	Tsukushin
**Vit**	Vitrin
**Vwa1-5**	5 von Willebrand factor A domain-containing proteins
**Vwc2/2l/e**	3 von Willebrand factor C domain-containing proteins
**VwD/E**	2 von Willebrand factor D and EGF domain proteins
**Zp1-4**	4 Zona pellucida glycoproteins
**Zp3r**	Zona pellucida 3 receptor
**Zpld1**	Zona pellucida-like domain-containing 1

Supplementary Table S3. Proteoglycans

Gene names	Protein names and synonyms
**INTRACELLULAR**	
Srgn	Serglycin
**CELL SURFACE**	
**Transmembrane**	
Sdc1-4	Syndecan, 1-4
Cspg4	NG2
Tgfbr3	Betaglycan/transforming growth factor, beta receptor III/
Ptprz1	Phosphacan/protein tyrosine phosphatase, receptor type Z, polypeptide 1
**GPI-Anchored**	
Gpc1-6	Glypican, 1-6
**PERICELLULAR**	
**Basement membrane**	
Hspg2	Heparan sulfate proteoglycan 2/perlecan
Agrn	Agrin
Col18a1	Collagen XVIII
Col15a1	Collagen XV
**EXTRACELLULAR**	
**Hyalectins/Lectins**	
Acan	Aggrecan
Bcan	Brevican
Vcan	Versican
Ncan	Neurocan

**Small leucine-rich proteoglycans (SLRPs)**	
**SLRPs - Canonical, Class 1**	
Bgn	Biglycan
Dcn	Decorin
Aspn	Asporin
Ecm2	Extracellular matrix protein 2, female organ and adipocyte specific
Ecmx	ECMX
**SLRPs - Canonical, Class 2**	
Fmod	Fibromodulin
Lum	Lumican
Kera	Keratocan
Omd	Osteomodulin/osteoadherin
Prelp	Proline arginine-rich end leucine-rich repeat/PRELP
**SLRPs - Canonical, Class 3**	
Epyc	Epiphycan
Ogn	Osteoglycin/mimecan
Optc	Opticin
**SLRPs – Non-canonical, Class 4**	
Chad	Chondroadherin
Chadl	Chondroadherin-like
Nyx	Nyctalopin
Tsku	Tsukushi
**SLRPs – Non-canonical, Class 5**	
Podn	Podocan
Podnl1	Podocan-like 1
**SPOCK**	
Spock1-3	Sparc/osteonectin, cwcv and kazal-like domains proteoglycan/testican 1-3
**Unclassified**	
Prg2-4	Proteoglycan, bone marrow 2-4
Impg1,2	Interphotoreceptor matrix proteoglycan 1,2
Esm1	Endocan/endothelial cell-specific molecule 1

Supplementary Table S4. Collagens

**Fibrillar (Type I, II, III, V, XI, XXIV, XXVIII)**	
Col1a1,2	Collagen type I alpha 1, 2 chain
Col2a1	Collagen type II alpha 1 chain
Col3a1	Collagen type III alpha 1 chain
Col5a1-3	Collagen type V alpha 1-3 chain
Col11a1,2	Collagen type XI alpha 1,2 chain
Col24a1	Collagen type XXIV alpha 1 chain
Col27a1	Collagen type XXVII alpha 1 chain
Col28a1	Collagen type XXVIII alpha 1 chain
**Network-forming (Type IV, VIII, X)**	
Col4a1-6	Collagen type IV alpha 1-6 chain
Col8a1	Collagen type VIII alpha 1 chain
Col10a1	Collagen type X alpha 1 chain
**Microfibril forming (Type VI)**	
Col6a1-6	Collagen type VI alpha 1-6 chain
**Anchoring fibrils (Type VII)**	
Col7a1	Collagen type VII alpha 1 chain
**FACIT (Fibril Associated Collagens with Interrupted Triple Helices (IX, XII, XIV, XVI, XIX, XX, XXI, XXII)**	
Col9a1,2,3	Collagen type IX alpha 1-3 chain
Col12a1	Collagen type XII alpha 1 chain
Col14a1	Collagen type XIV alpha 1 chain
Col15	Collagen type XV alpha 1 chain
Col16a1	Collagen type XVI alpha 1 chain
Col19a1	Collagen type XIX alpha 1 chain
Col20a1	Collagen type XX alpha 1 chain
Col21a1	Collagen type XXI alpha 1 chain
Col22a1	Collagen type XXII alpha 1 chain
**Short chain (Type VIII, X)**	
Col8a1,2	Collagen type VIII alpha 1,2 chain
Col10a1	Collagen type X alpha 1 chain
**Multiplexin (Multiple Triple Helix domains with Interruptions (Type XV, XVIII)**	
Col15a1	Collagen type XV alpha 1 chain
Col18a1	Collagen type XVIII alpha 1 chain
**MACIT (Membrane Associated Collagens with Interrupted Triple Helices) (Type XIII, XVII)**	
Col13a1	Collagen type XIII alpha 1 chain
Col17a1	Collagen type XVII alpha 1 chain
Col23a1	Collagen type XXIII alpha 1 chain
Col25a1	Collagen type XXV alpha 1 chain
